# A Data-Driven Based Response Reconstruction Method of Plate Structure with Conditional Generative Adversarial Network

**DOI:** 10.3390/s23156750

**Published:** 2023-07-28

**Authors:** He Zhang, Chengkan Xu, Jiqing Jiang, Jiangpeng Shu, Liangfeng Sun, Zhicheng Zhang

**Affiliations:** 1 College of Civil Engineering and Architecture, Zhejiang University, Hangzhou 310058, China; zjuzhanghe@zju.edu.cn (H.Z.); xck410@zju.edu.cn (C.X.); jpeshu@zju.edu.cn (J.S.); 2 Center for Balance Architecture, Zhejiang University, Hangzhou 310058, China; 3 Department of Civil Engineering, Zhejiang University City College, Hangzhou 310015, China; jiangjq@zucc.edu.cn; 4 The Architectural Design & Research Institute of Zhejiang University Co., Ltd., Hangzhou 310028, China

**Keywords:** structural-response reconstruction, conditional-generative adversarial network, deep learning, image processing

## Abstract

Structural-response reconstruction is of great importance to enrich monitoring data for better understanding of the structural operation status. In this paper, a data-driven based structural-response reconstruction approach by generating response data via a convolutional process is proposed. A conditional generative adversarial network (cGAN) is employed to establish the spatial relationship between the global and local response in the form of a response nephogram. In this way, the reconstruction process will be independent of the physical modeling of the engineering problem. The validation via experiment of a steel frame in the lab and an in situ bridge test reveals that the reconstructed responses are of high accuracy. Theoretical analysis shows that as the sensor quantity increases, reconstruction accuracy rises and remains when the optimal sensor arrangement is reached.

## 1. Introduction

With the increase of the service time of infrastructure, the safety assessment of civil infrastructure has become increasingly important, in which rich and comprehensive monitoring data are of great importance as the accordance for structural management and maintenance [1]. However, due to the cost associated with data acquisition, the responses of the structures in a few control sections could be recorded, but most of them are lost. Situations may exist in which the lost information is also critical for structural management and maintenance and needs to be obtained for a better understanding of the structural operation status. These structural responses that cannot be measured by sensors directly could be reconstructed with the measured responses [2,3].

A literature review of recent studies indicates there are primarily three types of methods for structural-response reconstruction. The first is the transmissibility concept-based approach proposed by Kammer [4], in which a matrix is defined to form the relationship between the measured and unknown responses. The transformation matrix in Kammer’s research consists of a set of inverse-system Markov parameters. Using this generalized transmissibility concept from Kammer’s work, S.S Law presented an approach for structural-response reconstruction in the frequency domain and applied the method in a seven-storey plane-frame structure [5]. In the same year, researchers expanded this method to the wavelet domain [6]. Using the unit impulse-response function, the relationship between the measured responses and the unknown response was formulated. The accuracy of this new approach proved to be higher in comparison to that of the frequency-domain approach. However, for applications using a transmissibility matrix, the information about the applied loading location is required. The second approach for response reconstruction is based on finite element modeling and the empirical-mode decomposition (EMD) of the structure, which is known as REMD [7]. In this method, the measured responses are decomposed into modal responses, which are used to compute unknown responses with the mode shape, which is obtained by a finite-element model. However, when the reconstruction problem involves closely spaced modes, the modes cannot be separated accurately, and the REMD method will be no longer valid. To overcome this shortcoming, this method is improved by dividing the structural-mode set into two sets: the closely spaced modes and the remaining modes. The responses of the closely spaced modal can be computed by the mode shapes and the rest of the modal response, which are measurable or can be calculated with mode shapes [8]. A Kalman filter-(KF) based method is the third method utilized in a structural-response reconstruction problem. Zhu et al. first used KF to solve the multitype-response reconstruction problem [9]. In their research, KF is implemented to measure the fused response with different types. X.H. Zhang extended Zhu’s work by taking the time-variant of the process and measurement-noise covariances into consideration and tested the proposed algorithm thorough numerical research on a simply supported overhanging steel beam [10]. The research works mentioned above provide great references with good accuracy for the reconstruction of structural response. 

The above-mentioned response reconstruction is implemented by solving equations of motions inversely, in which the simplification and assumptions of physical modeling may induce inevitable deviations. For instance, when the EMD-based reconstruction method is employed, if the closely spaced modes are involved, the modes cannot be separated accurately, and the REMD method is no longer valid. Meanwhile, the limitations imposed by the physical model may exist. According to the transmissibility matrix-based reconstruction method, the structural-response function is utilized to establish the relationship between the measured and reconstructed response; thus, the information of the loadings will be a necessary known condition. To address the above-mentioned shortcomings, the structural-response reconstruction method based on a data-driven mode is an alternative option. Due to the advancements in computer hardware and optimization algorithms, machine learning has been widely applied in various fields. An increasing number of data-driven structural mechanics applications have been proposed, such as structural damage identification [11,12] and external load identification [13,14]. Response reconstruction is another area where machine learning has found application. For instance, Shin [15] used a recurrent neural network (RNN) to reconstruct the dynamic response of a multi-DOF structure. Wu [16] compared the performance of the long-short-term memory (LSTM) and encoder of Transformer in reconstructing the flame nonlinear response. Lu [17] proposes a structural-acceleration response-reconstruction method based on bidirectional long-short-term memory (BiLSTM) networks and applied this method on a long-span cable-stayed bridge. However, in these studies, machine-learning algorithms are often regarded as regression models, used to reconstruct unknown responses based on known response information, which does not fully leverage the computational capabilities of machine learning. In fact, structural-response reconstruction can be viewed as a generative task, where the global structural response is reconstructed from known local responses holistically. As structural-response nephograms may offer both the information of the amplitude and the distribution simultaneously through images, this could be used to realize the response reconstruction from the perspective of response-nephogram processing. Meanwhile, the deep-learning method has been widely applied in image processing [18,19,20,21] and data reconstruction [22]. The conditional generative-adversarial network (cGAN) is one of the latest deep-learning algorithms and has been widely used in image processing because of its powerful image-generation ability. For example, cGAN is utilized to denoise astronomical images [23,24,25] and to identify cells from microscopic photos [26]. Owing to its good performance in the field of image processing, Farimani put forward a new data-driven paradigm for rapidly solving transportation problems [27]. With Farimani’s method, the solution of steady heat conduction and incompressible flow can be obtained directly by the condition without the control equation and the time and space iteration of traditional numerical simulation. cGAN is also employed to predict topology optimization under given boundary conditions [28]. With this method an approximate optimal network structure could be determined according to the pixel value and flexibility. The two investigations mentioned above achieved physical field simulation and structure design based on data-driven responses by means of image processing and provide references for reconstructing structural response in terms of generating structural-response nephograms. 

Different from the conventional method implemented by solving equations of motions inversely, a data-driven method based on cGAN is proposed for structural-response reconstruction. With this method, the global structural response could be reconstructed using the measured local response in the form of response nephogram. Thus, the errors due to the simplification and assumption of physical models in traditional methods will be avoided. cGAN is a kind of deep-learning method according to which the features of the measured response are extracted automatically with convolutional calculation. Meanwhile, in the framework of cGAN, the generative network is trained with a discriminal network in an adversarial manner. Benefiting from this adversarial-training strategy, the generative neural network can be optimized more efficiently. By treating structural-response reconstruction as a generative task and employing image-processing techniques, we can fully harness the potential of machine learning. This approach enables accurate capture of the complexity and spatial-distribution characteristics of structural responses, providing enhanced modeling and analysis capabilities for various engineering applications. With the optimized-generative network, the spatial relationship between global and local response is established in the form of response nephogram. The accuracy of the proposed method is verified by a steel frame and a steel box girder bridge. Furthermore, the effect of sensor quantity on reconstruction accuracy is studied. 

## 2. Methodology

As mentioned earlier, in most of the structural-response reconstruction processes, the precision of the methods usually relies on the accuracy of the physical modeling. However, with the increase in service time, the structural properties of engineering structure will deteriorate, and the physical model, which is established according to the original structural state, is no longer feasible in simulating the mechanical behavior of the structure. Meanwhile, the analysis based on a data-driven method with real-time structural-state information as an accordance may directly reflect the current service status of the structure. 

**Figure 1:**
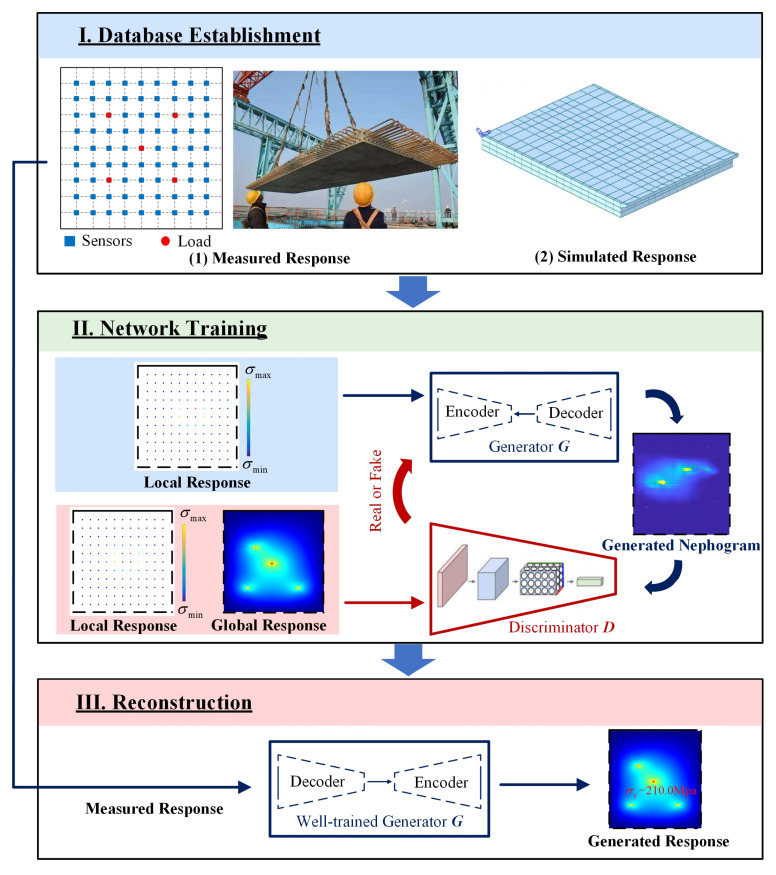
The overall workflow of the study.

Herein, a response-reconstruction method based on a deep-learning algorithm was developed. The schematic illustration of the overall workflow is depicted in Figure 1, in which the data-driven approach consists of three parts: (I) database establishment: a database will consist of the training dataset and the validation dataset, which are composed of structural-response data generated by the theoretical simulation method or the measured data respectively; (II) the cGAN training: cGAN architecture will be established to reflect the relationship between the global structural response and the local structural response and trained with the paired structural response locally and globally in the training dataset; the performance of the model will be validated with the data in the validation set; and (III) structural-response reconstruction: in this step, the measured local response will be input to the cGAN to construct the global response of the structure. Detailed descriptions of each part are as follows.

### 2.1. Database Establishment

The structural-response-reconstruction process is to construct the global structural response using the limited local response. In this process, the first step is to establish a database consisting of the global structural response paired with the local structural response. However, due to the economic cost or practical reasons, the number of sensors arranged on the structure for monitoring is usually limited, so that only the local structural response can be recorded with the global structural response missing. The accuracy of the database significantly affects the accuracy of machine-learning predictions. When a structure is newly constructed, it can be modelled with actual physical properties, and the simulated structural responses can be matched closely to the measured ones. However, as the structure ages, its physical performance tends to deteriorate, making it challenging to simulate real structural-response data through physical modeling. Nevertheless, during this period, long-term observations of structural-response information have been accumulated. Thus, the simulated data in the database can be replaced with actual measured data, enabling a data-driven approach for structural-response reconstruction based on measured data. Since the steel plate and the bridge utilized in this paper are brand new, responses of structures due to specific loadings are obtained by theoretical simulations to form a database, which will be used in network training. In addition, the measured data from practical engineering structures will be employed in validation.

In most cases, the load of the engineering structure during the service time is specific. For example, the bridge deck is usually subjected to vehicle load, etc., while the high-rise building, to wind load. This means that the form, size, and range of the loading on specific structures in service usually exhibits regularities. To make the database feasible to practical engineering structures, the loadings are usually arranged according to the actual situation in practical engineering during the service time. When possible loading conditions are fully considered in data generating, the database will be adequate for the structural-response reconstruction. 

**Figure 2:**
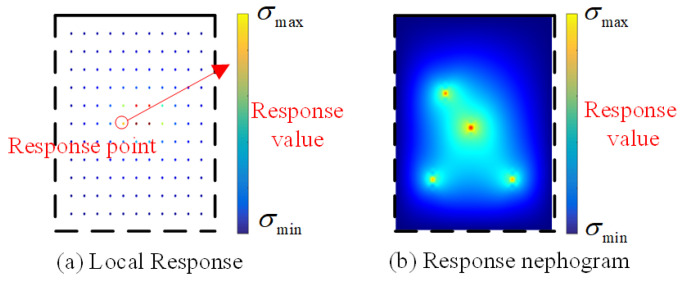
Sample with structural response.

The structural-response nephogram is the most intuitive means for descriptions of the structural response, which contains the most comprehensive information about responses all over the structure, including both the amplitude and the distributions. Thus, response nephograms are used as samples to establish databases for structural-response reconstruction. Taking the response of a plate due to a group of concentrated loads, for instance, the sample consists of a pair of nephograms, which represent the local structural response and the global structural response, respectively (Figure 2). The nephograms are defined according to the actual size of the plate. The four edge lines of the nephogram are used to represent the different boundary conditions: the dotted lines representing the simply supported edge, while the solid lines represent the fixed support edge. In the samples for the local response, a series of colorful dots are used to represent the local response, with the RGB value denoting the response value (Figure 2a). The size of samples is designed to 425∗425.

### 2.2. cGAN Training

After the database is prepared, a deep-learning neural network will be established and trained to build the relationship between the local response and the global response of the structure. The deep-learning neural network learns from data and recognizes patterns in a series of input and output data sets without any prior assumptions about their interrelations. The local structural response is used as input of the deep-learning neural network, and the output is the global response of structure.

Constructing the global structural response by limited local response is a generative task, and various similar applications have been successfully realized by cGAN [29,30,31]. cGAN is a machine-learning technique inspired by a two-player minimax game, which trains a pair of networks in an adversarial manner, known as generator *G* and discriminator *D* [32], whose training procedure is diagrammed in Figure 1(II). Generator *G* synthesizes a global-response nephogram according to the input local response, using convolution and deconvolution processes. In the convolution process, the feature of structural response is extracted and reconstructed into the global structural response using a deconvolution process. The discriminator *D* is trained to differentiate between the real sample and the generated nephogram.

**Figure 3:**
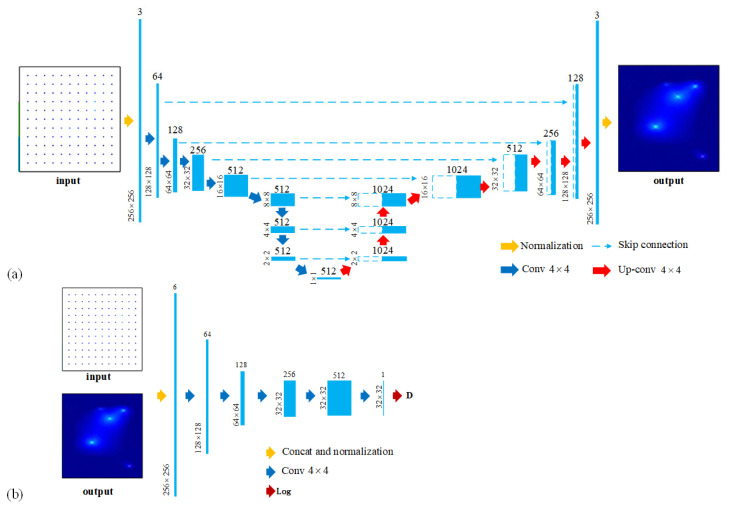
The architecture of the convolutional neural network (CNN) used in the cGAN (**a**) generator and (**b**) discriminator.

For the generator *G*, an encoder–decoder network (Figure 3a) is utilized to realize the convolution and deconvolution processes. In the convolutional neural-network (CNN) design, the convolution kernel size and the layer numbers are the two important factors affecting the network performance. The convolution kernel is the convolution matrix in the convolution and deconvolution processes. Large convolution kernels capture more information because of the bigger receptive field, but this will increase the computational work. Meanwhile, CNN extracts features through the convolution layer, and more layers mean the deeper features can be extracted. However, the increasing layer numbers may cause the calculated gradients of loss function to shrink to zero after numerous applications of the chain rule, known as the vanishing gradient. Thus, for both computational efficiency and reconstruction quality, the layer numbers and the kernel size of generator *G* are designed as Figure 3a, which is adjusted from Bittner et al. [33]. (See Appendix A.2 for the cGAN parameter selection.) In the first layer of G, the input sample for the local response is first normalized into regular data (256∗256). Then the normalized picture is processed by 8 convolutional operations using 4∗4 kernel and finally downsampled into a latent vector (512∗1). The process is then reversed by the decoder network (Figure 3). With a series of deconvolutional operations, the reduced vector representation is upsampled and resized into the inferred global-response nephogram. Seven skip connections are used to share information between the encoder and decoder. Discriminator *D* is designed to be a “patchGAN” classifier architecture with 5 convolutional layers (Figure 3b). The input to discriminator *D* is a concatenation of local structural responses, with the reconstructed structural response or the ground-truth structural response. After the combined input data pass through a series of convolutional layers and a fully connected layer, a probability that the input comes from will be produced.

In the training process of cGAN, the *D* will help *G* generate a more realistic global-response nephogram, and the nephograms generated by *G* will also be used as error samples for *D* to upgrade. In this adversarial manner, the generator *G* and discriminator *D* compete against each other and develop together. Lastly, the optimized *G* will be able to generate a response nephogram fitting to the distribution of the samples in the database. In order to realize this adversarial manner, a value function (Equation (1)) is proposed to train the generator *G* and discriminator *D* together. In the training process, the discriminator *D* is optimized by maximizing *V*(*D*,*G*), expressed as $\underset{D}{\max}V(D,G)$. Then the generator *G* is optimized by minimizing $\underset{D}{\max}V(D,G)$, expressed as $G*=\underset{G}{\min}\;\underset{D}{\max}V(D,G)$.

(1) $$\underset{G}{\min}\;\underset{D}{\max}V(D,G)=E_{x\sim p_{data}(x)}[\log(D(x|y))]+E_{x\sim p_{z}(z)}[\log(1-D(G(z|y)|y))]$$

### 2.3. Structural Response Reconstruction

After a well-trained generator *G* is obtained, it will be used to reconstruct the global structural response with the local response. The reconstructed result quality is quantitatively evaluated in terms of the relative error (*RE*), the structural similarity index measure (*SSIM*), and peak signal to noise ratio (*PSNR*). The *RE* of the peak response is expressed by the square norm number as:

(2) $$RE=\frac{{\mathrm{norm}}_{2}(\sigma_{i}-\sigma_{o})}{{\mathrm{norm}}_{2}(\sigma_{o})}$$

where $\sigma_{o}$ is the original peak response of the structure, and $\sigma_{i}$ is the reconstructed data.

*SSIM* is used to evaluate the structure similarity between the original and reconstructed images, which integrate luminance *l*, contrast *c*, and structure *s*. *SSIM* considers not only the value of individual pixels, but also the structural arrangement of these pixels. For the reconstructed response *x* and real response *y*, the *SSIM* is:

(3) SSIM(x,y)=[l(x,y)][c(x,y)][s(x,y)]

(4) $$[l(x,y)]=\frac{2\mu_{x}\mu_{y}+C_{1}}{\mu_{x}^{2}+\mu_{y}^{2}+C_{1}}$$

(5) $$[c(x,y)]=\frac{2\sigma_{x}\sigma_{y}+C_{2}}{\sigma_{x}^{2}+\sigma_{y}^{2}+C_{2}}$$

(6) $$[s(x,y)]=\frac{2\sigma_{xy}+C_{3}}{\sigma_{x}\sigma_{y}+C_{3}}$$

where *μ*_*x*_ and *μ*_*y*_ represent the mean of the reconstructed and original response, *σ*_*x*_ and *σ*_*y*_ denote the standard deviations, and *σ*_*xy*_ is the covariance of the two responses. To avoid a null denominator, *C*_1_, *C*_2_, and *C*_3_ are determined as positive constants. The value of the *SSIM* ranges from 0 to 1. The higher the value is, the more accurate the response reconstructed. Since each pixel value is treated equally in the *SSIM* calculation, *SSIM* is used to evaluate the accuracy of the overall distribution of the reconstructed response.

The *PSNR* is utilized to evaluate the reconstruction performance of noise suppression and is defined as a dimensionless metric expressed as:

(7) $$PSNR=10\cdot \log_{10}(\frac{MAX_{I}^{2}}{MSE})$$

(8) $$MSE=\frac{1}{mn}\sum_{i=0}^{m-1}\sum_{j=0}^{n-1}{\left[I(i,j)-K(i,j)\right]}^{2}$$

where *K* and *I* are the matrix representation of the reconstructed and original response. In the image, *m* and *n* denote the row and column number of pixels. *MAX_I_* represents the maximum pixel value of the image sample with the original response. The higher the value of the computed *PSNR*, the better the match between the reconstructed and original response. Different from *SSIM*, the maximum pixel value plays a more important role than other pixel values in the calculation of *PSNR*. Thus, the *PSNR* is utilized to evaluate the reconstruction performance on the peak-response distribution. 

## 3. Model Implementation Performance

A plate is a common substructure in civil engineering, such as a bridge deck and floor plate. Thus, a static loading experiment based on a steel plate was carried out to validate the feasibility of the proposed methodology. Furthermore, based on the validated model, the influence of the sensor number on the performance of the reconstruction procedure was analyzed. Finally, the proposed methodology was applied to reconstruct the structural response of a bridge.

### 3.1. Experimental Validation

**Figure 4:**
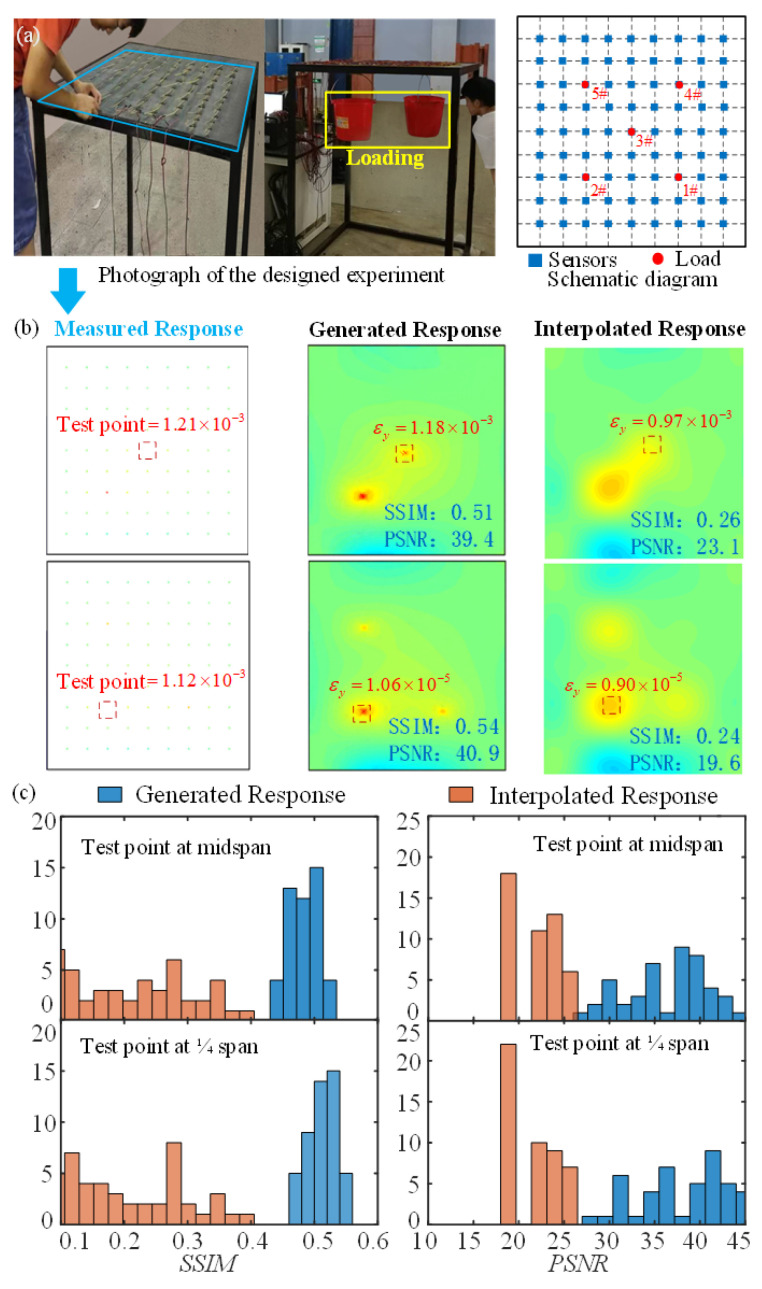
Brief experimental validation progress: (**a**) Photograph of the designed experiment; (**b**) The comparison of strain value between interpolation method, deep learning, and ground truth; (**c**) *SSIM* and *PSNR* of identification results using interpolation method and deep learning.

Four sides of the steel plate were fixed on a steel frame, and its physical parameters (Figure 4a) are listed in Table 1. The static concentrated loads were applied on the plate, consisting of two buckets of water. To realize the experimental validation of response reconstruction, 81 strain gauges were attached on the plate to capture the strain response. By adjusting the loading on the midspan or 1/4 span of plate (1#—5# in Figure 4a), 45 cases of experimental data were obtained, the loading condition of which is exhibited in Table A1 in the Appendix A.

According to the physical parameter and loading condition of the experiment, FEM simulation was utilized to generate samples with random-static concentrated loading. The brief FEM simulation method for the plate is presented in the Appendix. A training set was established with 2000 samples. During the network training process, the training set is also utilized as the validation set to evaluate model errors and facilitate real-time model assessment. Additionally, the test set comprises actual measurement data, including 45 cases of experimental data, and is applied to evaluate the performance of the trained cGAN model.

Two reconstruction examples are given at two positions, which are the 3# at the midspan of the plate and 2# at the 1/4 span of the plate (Figure 4b). The strain at the two positions is reconstructed with the remaining 80 strain using the deep-learning method (DL). The *RE* of the reconstructed strain using DL are 2.4% and 5.4%, which show much better agreement with the ground truth than the result using the linear interpolation method (IM), i.e., 19.8% and 19.6%, respectively (Figure 4b). The latter is the most commonly used response-reconstruction method. Furthermore, the distribution of *SSIM* and *PSNR* for the two methods is shown in Figure 4c and Table 2. These show that the average *SSIM* of the DL result is much higher than the IM result, and even the poorest *SSIM* of DL result is higher than the best result of IM. This suggests that DL performs better with reconstruction results than the interpolation method on the overall strain-response distribution. Meanwhile, the peak strain-response distribution reconstructed by DL is more accurate than IM, according to the comparison of the *PSNR* result. All three values indicate that the proposed DL method could successfully reconstruct the structural response.

### 3.2. Effect of Sensor Quantity

In practical engineering applications, it is uneconomical to mount a dense array of sensors. The response reconstruction may help to reduce the monitoring cost by reducing the sensor quantity. In considering both the accuracy and economy in the reconstruction process, the influence of the sensor quantity was studied.

**Figure 5:**
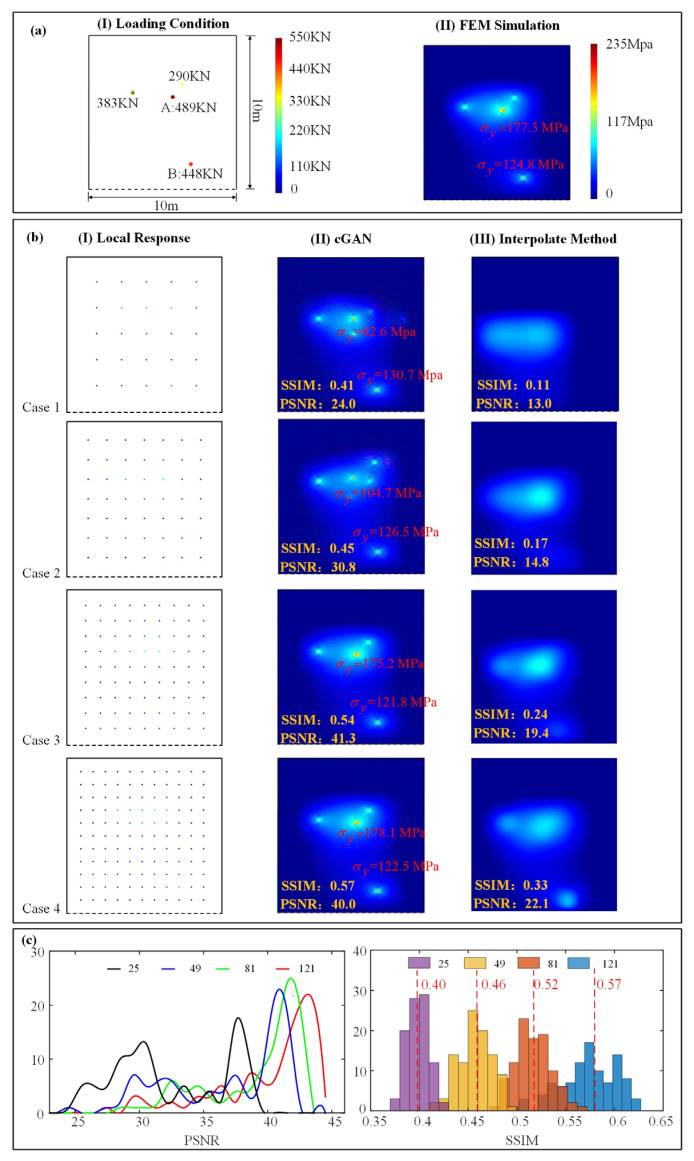
Reconstruction results with different sensor quantity: (**a**) loading condition on the plate and the corresponding global stress-response nephogram; (**b**) the reconstructed stress nephogram with different sensor quantity; (**c**) statistic comparison of the *SSIM* and *PSNR* with different sensor quantity.

The local strain responses of the plate (Figure 5b(I)) were obtained from FEM simulation (Figure 5a(II)), with the colorful spots presenting the local strain-response value, and the global strain responses were reconstructed through the well-trained cGAN (Figure 5b(II)). Four sensor arrays with different sensor quantities were arranged here to study their influence on the accuracy of reconstruction results. To evaluate the reconstruction performance due to different sensor quantities, the reconstructed stress values at the location where two concentrated loads were applied are marked out in the generated nephograms (see A and B in Figure 5a(I)). In the first two cases, the reconstruction result is obviously inconsistent with the ground truth. In the upper half region of the global stress nephograms, four stress peaks are observed, while only three loads were applied on the region in reality. As the sensors’ number increases to 81 in case III, the reconstruction accuracy is significantly raised. Intuitively, the qualities of the reconstructed stress are similar with ground-truth nephograms The *RE* of the reconstructed stress values at points A and B decrease to 1.1% and 2.4%, and the stress distribution of the reconstructed results also reach a high level (*SSIM* = 0.54 and *PSNR* = 41.3). While the sensor quantity rises to 121, the reconstruction quality improves slightly (*RE* = 1.8%, *SSIM* = 0.57 and *PSNR* = 40.0). Hence, herein the optimal value of the sensor quantity is 81 for the best accuracy and economy. In comparison, the corresponding interpolated results are exhibited. The comparison shows that the structural responses reconstructed by cGAN are far more accurate than the interpolated results. Concretely, even with 121 sensors, the accuracy of the interpolated result is worse than the result of cGAN reconstruction with 25 sensors. To study the stability of the response-reconstruction method due to different sensor quantities statistically, one hundred validation samples were reconstructed. The statistical results of their *SSIM* and *PSNR* agreed with the former conclusion, as shown in Figure 5c. 

### 3.3. Engineering Applications

**Figure 6:**
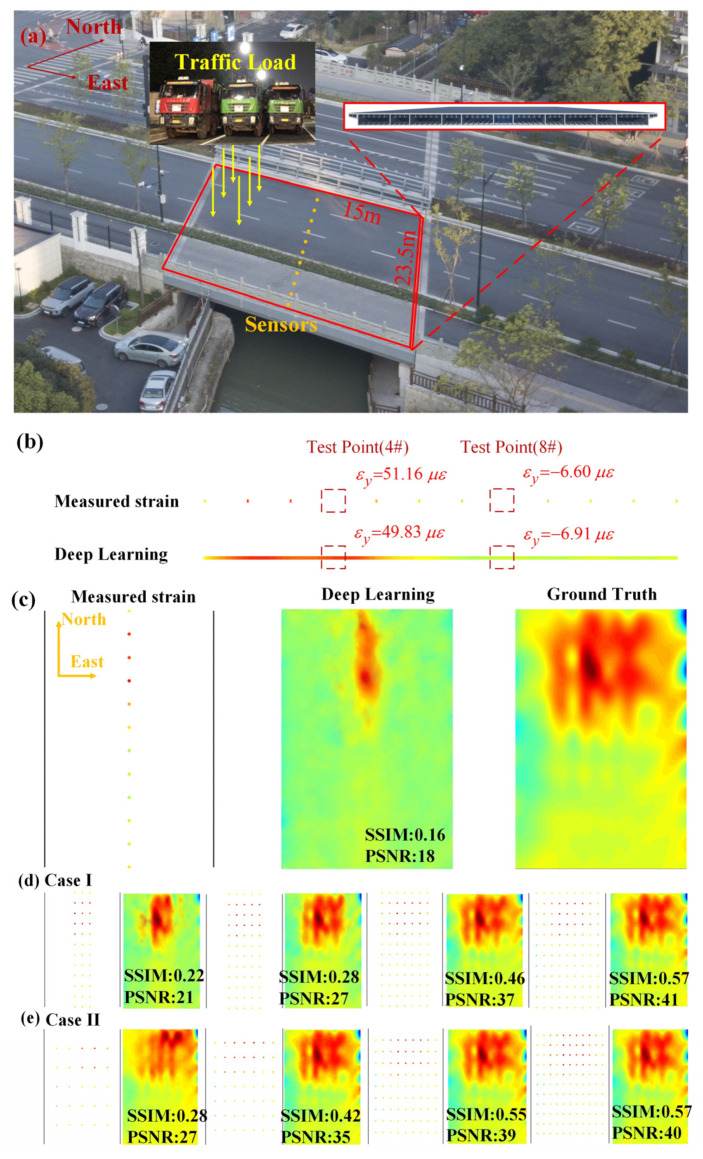
Brief test validation progress in practical engineering application: (**a**) photograph of the Huanzhen Bridge; (**b**) the reconstruction result on a single section; (**c**) the reconstructed global-response nephogram of bridge deck; (**d**,**e**) the global-response nephogram reconstructed with two- sensor arrangement.

A simply supported multi-cell steel box girder bridge (Figure 6a) was taken as a case to validate the feasibility of this method in a practical engineering application. The bridge is located in Hangzhou with the name of Huanzhen Bridge. The thicknesses of the top, bottom and web plates of the girder cell are 16 mm, 20 mm, and 12 mm, respectively. In the in situ site test, the bridge was exerted by a four-axle heavy truck with weight of 400 KN, with strain responses measured by 12 strain gauges attached at the bottom of the bridge in midspan. By adjusting the location of the truck, 200 samples were simulated for the network training of cGAN. Using the well-trained cGAN, the strain responses at the two measuring points (4# and 8#) were reconstructed with the strain responses from the remaining ten strain gauges (Figure 6b). The reconstruction results agreed reasonably well with the measured value. Further, the global-strain nephogram was reconstructed using all the 12 measured strain data (Figure 6c). In Figure 6c, the reconstructed strain responses in the neighborhood of the sensors are very close to the ground-truth; otherwise the reconstruction precision would not be good. 

To obtain a response nephogram of higher quality, the sensor array is assumed to be expanded from one column of 12 measuring points to 3, 5, 7, and 9 columns in numerical simulations. As shown in Figure 6d, when the sensor quantity reaches three columns, it becomes possible to fully reconstruct the stress concentration points. Furthermore, as the number of sensor array columns increases, the reconstructed strain nephogram expands gradually from the mid-span to the supports and becomes clearer. This expansion of the reconstructed region signifies an improvement in the effectiveness of the sensor array. The results of PSNR and SSIM indicate that the improvement in reconstruction accuracy is relatively small when the sensor columns are increased from 7 to 9 compared to the improvement from 5 to 7 columns. This observation can be attributed to the loading vehicle’s positioning at the mid-span while the supports are fixed, resulting in relatively smaller strains near the supports. 

The former sensor arrangement with one column of 12 sensors results in a dense distribution at the mid-span and a sparse distribution at the edges, which is evidently not cost-effective. In order to find a more cost-effective sensor arrangement, we investigated the reconstruction effects of a uniformly distributed sensor arrangement, as shown in Figure 6e. It can be observed that the quality of the reconstructed strain field improves significantly as the sensor arrangement transitions from 3 × 3 to 7 × 7. However, the improvement becomes relatively smaller when the transition is from 7 × 7 to 9 × 9. Therefore, the sensor arrangement with 49 sensors is considered the most cost-effective option.

## 4. Conclusions

The structural-response reconstruction is of great importance to enrich monitoring data for better understanding of the structural-operation status. Different from the conventional method implemented by solving equations of motions inversely, this paper proposed a data-driven method based on cGAN for structural-response reconstruction. Thus, the errors due to the simplification and assumption of physical models in traditional methods will be avoided. It is important to note that, although in this study we trained the network using simulated data and validated it using test data, the proposed response-reconstruction framework is applicable to both simulated and test data. When a structure is newly constructed, modeling it accurately with physical properties allows for the simulation of structural responses that closely match the measured results. However, as the structure ages, its physical performance deteriorates, making it challenging to simulate real structural-response data through physical modeling. Nevertheless, during this period, valuable long-term observations of structural-response information have been accumulated. Thus, the simulated data in the database can be replaced with actual measured data, enabling a data-driven approach for structural-response reconstruction based on measured data. This approach enables a data-driven methodology for structural-response reconstruction based on measured data.

The conclusions are summarized as follows,

(1) In the proposed method, cGAN is employed to establish the spatial relationship between the global and local response in the form of a response nephogram, which consists of generative network *G* and discriminal network *D*. During the image processing by generative network *G*, the features of the measured response are extracted with convolutional calculation and deconvoluted into a global structural-response nephogram, while discriminal network *D* is designed as a binary-classification network to differentiate between the real and the generated nephogram. By the adversarial training strategy between generator *G* and discriminator *D*, cGAN is optimized more effectively.
(2) Since a plate is a commonly used substructure in civil engineering, an experiment with a steel plate is carried out to validate the feasibility of the proposed methodology. The accuracy of the method is quantitatively evaluated in terms of *RE*, *SSIM* and *PSNR*, which indicate that the proposed method could successfully reconstruct the global structural response according to local ones.
(3) The influence of the sensor quantity on reconstruction performance is studied based on specific statistical analysis. Results show that the reconstruction accuracy rises as the sensor quantity increases and remains when the optimal sensor arrangement is reached.
(4) An Engineering application based on a steel box girder bridge is carried out. The reconstruction results agree reasonably well with the measured value, and the reconstruction precision rises as the distance from the sensor decreases. Ultimately, the cost-effective sensor arrangement for the bridge is obtained by comparing the reconstruction performance of two sensor arrangements.
(5) While the primary object of this study is plate structure, the demonstrated reconstruction examples of responses in plate cross-sections indicate the potential for extending the application of cGAN-based structural-response reconstruction methods to other sub-structures such as beams and columns.
(6) The reconstruction of structural response is essentially a problem in inverse mechanics, and in this study, the cGAN-based method has achieved promising results. In future research, it is possible to extend this method to other classical inverse mechanics problems, such as load inversion and damage identification. By applying similar principles and techniques, the proposed approach can be adapted and further developed to address these challenges. This expansion of the method’s applicability will contribute to the broader field of inverse mechanics and enhance our understanding of structural behavior and integrity assessment.

## Figures and Tables

**Table 1:** The physical parameters of the steel plate.

Physical Parameters	Value
Length (*a*), Width (*b*)	1 m
Thickness	5 mm
Young’s modulus (*E*)	210 GPa
Poisson’s ratio (ν)	0.3

**Table 2:** The *SSIM* and *PSNR* of reconstruction result.

Test Point	Midspan		1/4 Span	
	DL	IM	DL	IM
*SSIM*	0.48 ± 0.05	0.21 ± 0.19	0.51 ± 0.05	0.20 ± 0.19
*PSNR*	36.2 ± 10.2	21.8 ± 4.3	32.5 ± 10.4	21.4 ± 4.7

**Figure A1:**
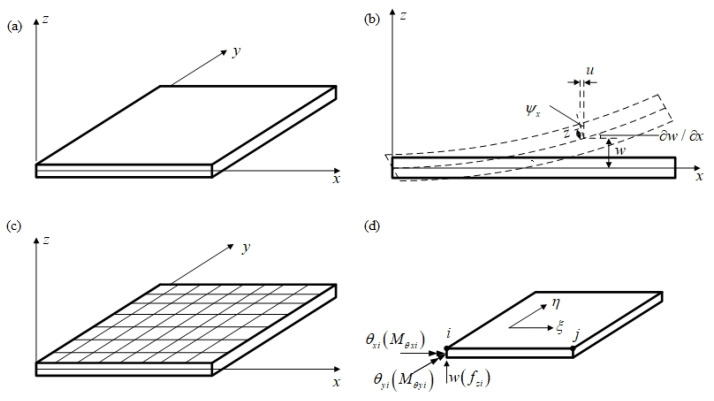
Finite element diagram of plate. (**a**) plate structure. (**b**) deformation illustration. (**c**) division of plate elements. (**d**) plate elements.

**Figure A2:**
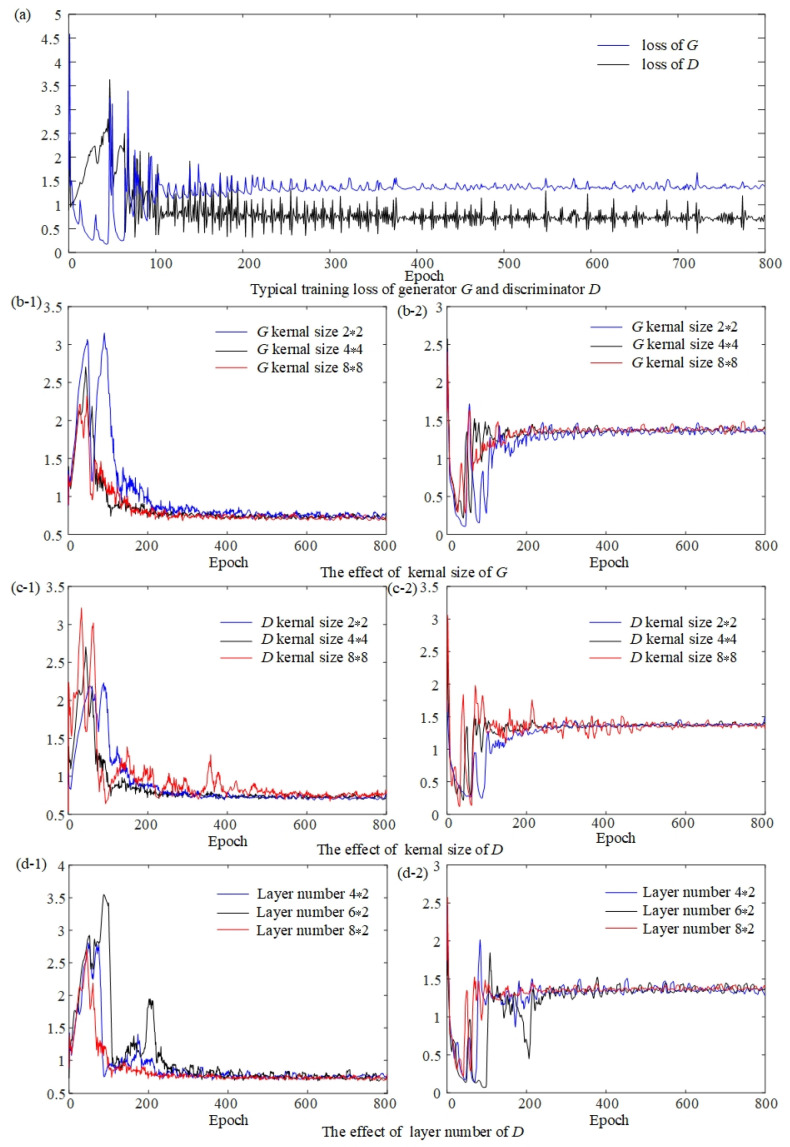
The training process of generator *G* and discriminator *D* with different network parameters.

**Figure A3:**
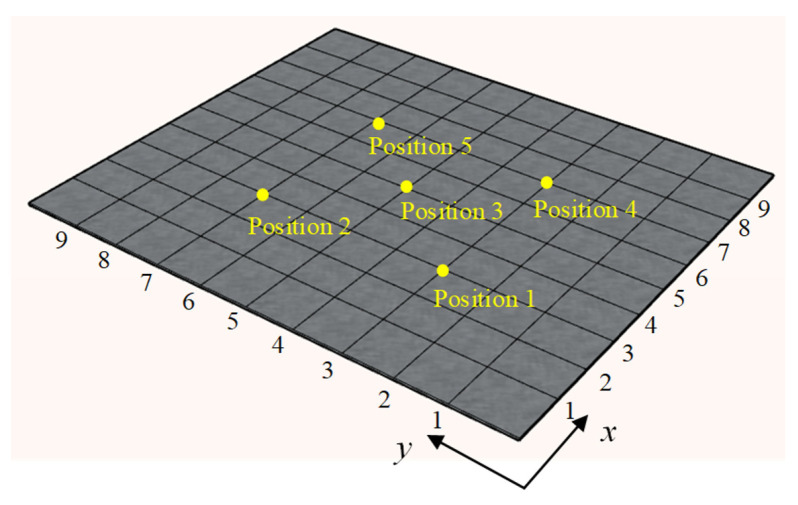
Schematic diagram of plate and loading position.

## Data Availability

The test data is unavailable due to privacy.

## Appendix A

### Appendix A.1. FEM Method for Plate

The finite element method for plate structure is as follows. As the neutral axis (strain axis $\varepsilon_{x}=\varepsilon_{y}=\varepsilon_{z}=0$) of the plate is located on the *xy* plane, specifically at *z* = 0, as shown in Figure A1, then according to the assumption of straight normal lines, the normal lines perpendicular to the neutral axis before deformation remain straight lines after deformation, but they may not be perpendicular to the deformed neutral axis. The rotations of the normal lines around the *y* and *x* axes after deformation are denoted as $\psi_{x}$ and $\psi_{y}$, respectively, as shown in Figure A1b. Assuming that the lateral displacement *w* of the plate remains constant along the thickness direction, i.e., $\varepsilon_{z}=0$, the displacement and strain at any point at a distance *z* from the neutral axis can be expressed as,

(A1) $$\begin{array}{l} \begin{array}{cccc} u=-z\psi_{x} & v=-z\psi_{y} & \varepsilon_{x}=-z\psi_{x\prime x} & \varepsilon_{y}=-z\psi_{y\prime y} \end{array}\\ \begin{array}{ccc} \gamma_{xy}=-z\left(\psi_{x\prime y}+\psi_{y\prime x}\right) & \gamma_{yz}=w_{\prime y}-\psi_{y} & \gamma_{xz}=w_{\prime x}-\psi_{x} \end{array} \end{array}$$

In this case, when the plate is sufficiently thin, Kirchhoff’s plate theory can be applied, which neglects the transverse shear deformation, $\gamma_{yz}=\gamma_{xz}=0$. The strain can then be expressed as the curvature of the deflection, given by,

(A2) $$\varepsilon =\left\{\begin{array}{c} \varepsilon_{x}\\ \varepsilon_{y}\\ \gamma_{xy} \end{array}\right\}=-z\left\{\begin{array}{c} w_{\prime xx}\\ w_{\prime yy}\\ 2w_{\prime xy} \end{array}\right\}$$

Discretizing the plate structure into rectangular elements, as shown in Figure A1c, the nodal displacement vector can be represented as Equation (A3), and the corresponding nodal force vector is given as Equation (A4).

(A3) $$\delta_{i}=\left\{\begin{array}{c} w_{i}\\ \theta_{xi}\\ \theta_{yi} \end{array}\right\}=\left\{\begin{array}{c} w_{i}\\ w_{\prime yi}\\ -w_{\prime xi} \end{array}\right\}$$

(A4) $$f_{i}=\left\{\begin{array}{c} f_{zi}\\ M_{\theta xi}\\ M_{\theta yi} \end{array}\right\}$$

Meanwhile, local coordinates Oξη are established for each plate element. The local coordinates of the plate element are related to the global coordinates of the plate structure as follows,

(A5) $$x=x_{0}+a\xi ,y=y_{0}+b\eta$$

where $x_{0}$ and $y_{0}$ are the global coordinates of the center of the plate element, and a and b represent half of the lengths of the plate element along the ξ and η directions, respectively.

Furthermore, the deflection w can be expressed using the local coordinates ξ and η, as follows.

(A6) $$\begin{array}{l} w=\alpha_{1}+\alpha_{2}\xi +\alpha_{3}\eta +\alpha_{4}\xi^{2}+\alpha_{5}\xi \eta +\alpha_{6}\eta^{2}+\alpha_{7}\eta^{3}\\ +\alpha_{8}\xi^{3}+\alpha_{9}\xi \eta^{2}+\alpha_{10}\xi^{2}\eta +\alpha_{11}\xi \eta^{3}+\alpha_{12}\xi^{3}\eta \end{array}$$

Therefore, using the deflection w as an intermediate variable, the relationship between strain ε and nodal displacements δ can be established as follows:

(A7) $$\varepsilon =Β\delta^{e}$$

By applying the variational principle, the stiffness matrix for the plate element ${Κ}_{ij}$ can be obtained.

(A8) $${Κ}_{ij}=∭z^{2}B_{i}^{T}DB_{j}dxdydz=\frac{h^{3}}{12}\int_{-1}^{1}\int_{-1}^{1}B_{i}^{T}DB_{j}abd\xi d\eta$$

Finally, by assembling the element stiffness matrix ${Κ}_{ij}$ into the global stiffness matrix Κ, and the nodal load vector $f_{i}$ into the global load vector f, we can obtain the overall displacement of the plate structure as follows:

(A9) δ=Κ\f

### Appendix A.2. cGAN Parameter Selection

The loss function of a Generative Adversarial Network (GAN) consists of two components: the generator’s loss and the discriminator’s loss.

The generator’s loss function primarily measures the discrepancy between the generated samples and the real samples. Typically, the generator’s loss is calculated as the cross-entropy between the output of the discriminator when evaluating the generated samples and the labels of real samples. The goal of the generator is to minimize this loss, aiming to generate samples that closely resemble the real samples.

The discriminator’s loss function is used to assess the discriminator’s ability to differentiate between the generated samples and the real samples. It comprises two parts: one part is the cross-entropy between the output of the discriminator when evaluating the generated samples and their labels, and the other part is the cross-entropy between the output of the discriminator when evaluating the real samples and their labels. The objective of the discriminator is to minimize this loss, enhancing its ability to discriminate between real and generated samples.

Unlike other neural networks such as CNNs, BP, and RNNs, in which a lower loss value indicates better network performance, the evaluation of GAN training effectiveness is not solely based on the magnitude of the loss value. Instead, the stability of the loss value is considered. In GAN training, the generator and discriminator are engaged in an adversarial competition. The generator aims to generate samples that resemble the real samples, while the discriminator aims to accurately distinguish between real and generated samples. As a result, the loss values of the generator and discriminator may oscillate during training. Rather than focusing on achieving a loss value close to zero, the stability of the loss values is important in GAN training. When the loss values of both the generator and discriminator stabilize, it indicates that the generator has learned to generate realistic samples and the discriminator has learned to effectively discriminate between real and generated samples. This balance between the generator and discriminator is a key indicator of the training effectiveness of a GAN. Therefore, we analyzed the effect of different convolutional kernels and the number of convolutional layers in *G* on the optimization speed of cGAN by examining the stability of the loss values for *G* and *D*.

Figure A2a demonstrates the typical training loss of cGAN, where *G* and *D* gradually stabilize after adversarial training. Faster stabilization indicates better performance. Thus, we compared the stability of *G* and *D* loss and analyzed the impact of different convolutional kernels and the number of convolutional layers in G on the optimization speed of cGAN. As shown in Figure A2b, the convolutional kernel size of 4∗4 for *G* exhibits the fastest stabilization, making it the most suitable choice. Similarly, for *D*, a convolutional kernel size of 4∗4 (Figure A2c) is also optimal. Among different configurations of *G* convolutional layers, the cGAN model with 8 convolutional layers and 8 deconvolutional layers shows the fastest stability. Therefore, we selected a cGAN network with 4∗4 convolutional kernels and 8 convolutional layers and 8 deconvolutional layers. However, it should be noted that this analysis is based on the stability of network training, and further performance validation is necessary by considering specific sample cases.

For reference, the learning rate in our study was set to 0.0002, with a batch size of 1. The number of iterations ranged from 500 to 800. These settings were determined based on experimentation and balancing computational efficiency with achieving desired training results.

### Appendix A.3. The Loading Condition of the Experiment

The plate and loading position of the in-lab experiment is present as Figure A3, with the corresponding loading condition summarized in Table A1.

**Table A1:** The loading condition of the experiment.

Condition	Load Value (N)				
	Position 1	Position 2	Position 3	Position 4	Position 5
1	100	50			
2	100		50		
3	100			50	
4	100				50
5	50	100			
6		100	50		
7		100		50	
8		100			50
9	50		100		
10		50	100		
11			100	50	
12			100		50
13	50			100	
14		50		100	
15			50	100	
16				100	50
17	50				100
18		50			100
19			50		100
20				50	100
21	100	100			
22	100		100		
23	100			100	
24	100				100
25		100	100		
26		100		100	
27		100			100
28			100	100	
29			100		100
30				100	100
31	100	100	100		
32	100	100		100	
33	100	100			100
34	100		100	100	
35	100		100		100
36	100			100	100
37		100	100	100	
38		100	100		100
39		100		100	100
40			100	100	100
41	100	100	100	100	
42	100	100	100		100
43	100	100		100	100
44	100		100	100	100
45		100	100	100	100
